# Anatomical and molecular development of the human primary visual cortex

**DOI:** 10.3389/fncel.2024.1427515

**Published:** 2024-09-30

**Authors:** Kathryn M. Murphy, Leanne Monteiro

**Affiliations:** ^1^McMaster Neuroscience Graduate Program, McMaster University, Hamilton, ON, Canada; ^2^Department of Psychology, Neuroscience and Behavior, McMaster University, Hamilton, ON, Canada

**Keywords:** human, visual cortex, V1, development, histology, neuroanatomy, molecular, plasticity

## Abstract

The human primary visual cortex (V1) development is pivotal to understanding cortical maturation and neuroplasticity. Theories on V1 development range from early maturation models, which emphasize the early peak of synapses in infancy, to those suggesting an extended developmental timeline where key plasticity mechanisms continue to mature well into adulthood. Classic histological approaches have supported early development, while recent molecular studies highlight prolonged or multiple windows of plasticity, indicating that V1 remains susceptible to experience-dependent modifications beyond childhood. This review consolidates findings from both anatomical and molecular studies, tracing the development of V1 from prenatal stages through aging. The evidence reveals that human V1 develops across multiple timescales, with some aspects maturing early and others gradually changing across the lifespan. Reflecting on Cajal’s early work, this review underscores the importance of methodological advancements in revealing the intricate details of V1’s development.

## Introduction

The development of the human visual cortex (V1) has been a central theme in the two leading theories about cortical development: the system-by-system cascade, where V1 develops early (Huttenlocher and Dabholkar, 1997) and the integrated network, where V1 develops over a protracted period (Lidow et al., 1991). Classic histological investigations of V1 in infants, such as synapse counts, point to early development of human V1. Moreover, these studies suggest that the peak in synapse counts define the window of highest neuroplasticity in the human cortex (Huttenlocher, 1999; Petanjek et al., 2023). In contrast, studies using a variety of molecular approaches have shown that many of the mechanisms that regulate experience-dependent plasticity continue to mature past childhood into the second and third decades of life, suggesting that V1 has prolonged or multiple plasticity windows (Siu and Murphy, 2018). Furthermore, the protracted development of human V1 could provide a longer window for visual experience to shape its function and support plasticity-based treatments for visual disorders (Castaldi et al., 2020; Thompson et al., 2023).

In humans, more than 20 cortical areas process visual information, so the development of V1 can have a ripple effect on many cortical areas and perceptual functions. Animal models, especially those using mice, continue to reveal the unique impact of vision on the cellular landscape of the cortex (Chen et al., 2024). However, studying human V1 development using postmortem tissue is challenging because brain banks have limited cases at certain ages, and the samples are often delicate and tricky to work with. Over the past century, a growing body of anatomical and molecular research has used those rare and valuable samples to create a picture of how human V1 changes across the lifespan. This review consolidates the insights gained from classic cellular histology and modern molecular techniques, offering an overview of human V1 development covering prenatal and postnatal development and highlighting molecular mechanisms that regulate experience-dependent plasticity in V1.

### Prenatal development of human V1

**FIGURE 1:**
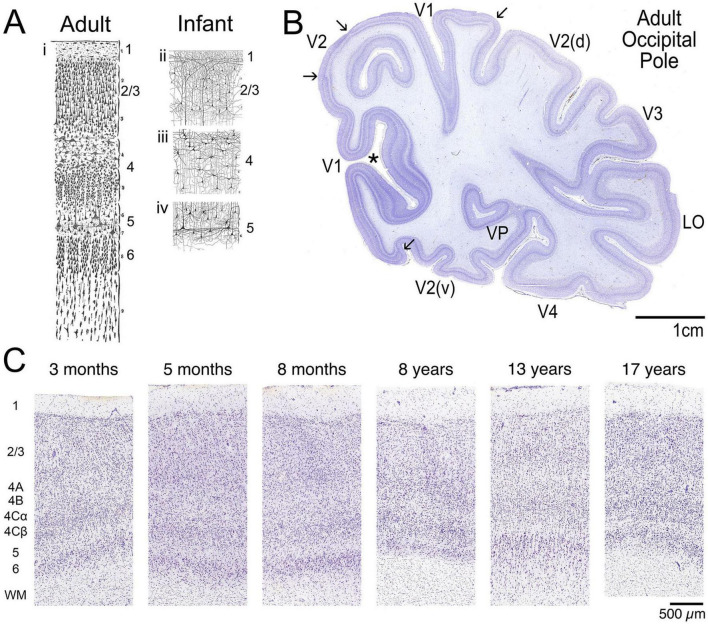
Human V1 cytoarchitecture. **(A)** Drawings by Santiago Ramón y Cajal showing the cytoarchitecture of human V1 through the full thickness of a Nissl stained section from (i) adult V1 and (ii-iv) smaller examples from Golgi stained sections of cells in layers 2/3, 4 and 5 from the infant V1. Even in the infant, the apical dendrites of pyramidal cells extend vertically across layers. Adapted from Figures 1, 4, 9 and 20 from the book “Comparative study of the sensory areas of the human cortex”, pages 314, 325, 337 and 353 (Cajal, 1899). Note: the original drawings do not include scale bars so these examples could not be matched for magnification; the book is available at: https://archive.org/details/comparativestud00cajagoog/. **(B)** Nissl-stained through the occipital pole of an adult human showing the unique laminar pattern of V1 and the different appearance of the neighboring extra-striate areas. In addition, variation in thickness of V1 is apparent through the depth of the calcarine fissure (asterisk). **(C)** The postnatal appearance of human V1 is shown in a series of Nissl-stained sections from 3 months to 17 years of age. All 6 layers are visible postnatally. Layer 1 is the outermost layer and is lightly stained because it has few cell bodies. Layers 2 & 3 have many darkly stained cells including smaller pyramidal at the top of layer 2 to medium sized pyramidal cells in layer 3. Layer 4 is the recipient zone for thalamocortical afferents and has 3 main sublaminae with larger stellate cells in 4A and smaller stellate cells, often called granule cells, through layers 4B, 4Cα and 4Cβ that give rise to the appearance of the stria of Gennari. Layer 5 appears lighter than layer 4 but is distinct because it has a band of large subcortically projecting pyramidal cells. Layer 6 has medium sized pyramidal cells at the top and a dense combination of smaller cells with a range of shapes in the deeper portion of this layer. The white matter under layer 6 has tiny round cells, the oligodendrocytes that form myelin, ensheathing the axons. In one case, the 13-year-old, some interstitial cells are visible in the white matter (Kostovic and Rakic, 1980, 1990; Judaš et al., 2010). Images of the Nissl-stained sections are from: © 2010 Allen Institute for Brain Science. Allen Human Brain Atlas. Available from: atlas.brain-map.org and © 2010 Allen Institute for Brain Science. BrainSpan Reference Atlas. Available from: www.brainspan.org/static/atlas.

The detailed cytoarchitecture of the human primary visual cortex (V1) has been known since the late 19th century, as shown by Cajal’s histological slides and drawing of neurons in V1 (Figure 1A; Cajal, 1899). Cajal’s precise drawings of human V1 using Nissl and Golgi stained sections primarily from infants led him to conclude that:

*The visual cortex of man and gyrencephalous mammals possesses a special structure very different from that of any other cortical area (Cajal, 1899)*.

The early stages of human cortical development have been reviewed in detail (Molnár et al., 2019), so here we highlight the development of V1. Changes in the first trimester of prenatal life include extending ventricular and outer radial glia fibers to the pial surface of the developing cortical plate (Bystron et al., 2008; Nowakowski et al., 2016; Arellano et al., 2021). The maturation of radial glia cells projecting to V1 lags behind those to the prefrontal cortex by about 3 weeks (Nowakowski et al., 2017). Next, neurogenesis starts (Bhardwaj et al., 2006; Clowry et al., 2010) along with peak levels of neurogenesis-specific gene expression (Zhu et al., 2018). Newly differentiated neurons migrate from the ventricular zone into the developing cortical plate along radial glia fibers (Rakic, 1972), and the cortical layers mature in an inside-out order: the neurons that will form layer 6 are born first, followed by neurons destined for layers 5 through 2 (Rakic, 1974). The supragranular neurons (layers 2/3) use outer radial glia to migrate through the cortical plate (Nowakowski et al., 2016). During the peak of neurogenesis, maturing excitatory neurons already express a rudimentary set of V1-specific marker genes (Nowakowski et al., 2017).

Throughout the second trimester, the morphological structure of the neurons begins to develop, and thalamocortical afferents grow into the transient subplate between 22 and 26 weeks of gestation and wait briefly before entering the cortical plate (Kostovic and Rakic, 1980). The cortical layers start to differentiate before thalamic afferents invade the cortical plate with deep layers apparent by 19 weeks of gestation (Takashima et al., 1980; Flower, 1985; Burkhalter, 1993; Burkhalter et al., 1993), followed by layer IV at 28 weeks and the superficial layers II/III at 28–35 weeks (Takashima et al., 1980). By the 24–25th week of gestation, the thalamocortical afferents begin to mature and express neuronal markers (Godovalova, 2010). Just a few weeks later (29th–31st weeks), functional connectivity between the thalamus and V1 begins to strengthen (Taymourtash et al., 2022), starting the period when thalamocortical activity can influence the development of V1 and a sharp increase in genes specific to neuronal differentiation (Zhu et al., 2018).

Both excitatory glutamatergic and inhibitory GABAergic neurons are found prenatally in the developing cortical plate (Yan et al., 1992; Andersen et al., 1995; Retz et al., 1996). Many of those cells, as well as synapses and thalamocortical afferents, form a transient arrangement in the subplate zone during the second and third trimesters (Kostović and Judaš, 2007). For example, dense parvalbumin (PV+) labeling is prominent in the subplate in the second trimester and, by birth, has shifted to a cortical laminar arrangement of PV+ inhibitory interneurons (Cao et al., 1996) that continues to mature until adolescence (Letinic and Kostovic, 1998).

In addition to neurons, markers for glia are found prenatally below or in the developing cortical plate of V1. Early in the second trimester, microglia cluster in the immature white matter among the visual radiations and by the middle of the second trimester are found throughout the thickness of the developing V1 (Monier et al., 2006, 2007). Astrocytes are also found in the developing V1 by the middle of the second trimester (Honig et al., 1996; Godovalova, 2010), and astrocyte-specific gene expression in human V1 continues to increase until early childhood (Zhu et al., 2018). Myelinating oligodendrocytes begin accumulating in the white matter below V1 but do not infiltrate the cortical plate before birth (Kostović et al., 2014), leaving V1 with the translucent appearance characteristic of the neonatal cortex.

The thickness of V1 grows from 100 μm to nearly 2 mm through the prenatal period and continues to grow postnatally (Zilles et al., 1986; Molnár et al., 2019). The volume and surface area of V1 has the greatest period of growth postnatally as dendrites and spines on pyramidal neurons contribute to the increased thickness of the cortex, with spines reaching a peak at 5 months before falling to adult levels by 2 years of age (Takashima et al., 1980; Becker et al., 1984; Michel and Garey, 1984). However, V1 does not have a uniform thickness and the gyral and sulcal pattern, especially deep in the calcarine fissure, leads to large variations in thickness. Furthermore, host factors such as sex and socio-economic status are known to affect the thickness of the developing cortex (Sowell et al., 2007; Mackey et al., 2015; Gennatas et al., 2017; Leonard et al., 2019; Alnćs et al., 2020; Tooley et al., 2021).

Other anatomical features, such as columnar patterns and horizontal connections, first appear in human V1 prenatally and continue to develop postnatally. Patchy cytochrome oxidase staining is visible prenatally, with distinct blobs apparent at birth (Burkhalter et al., 1993; Wong-Riley et al., 1993). The early pattern of blobs likely reflects the patchy arrangement and spontaneous activity of thalamocortical afferents projecting into the cortex. The plexus of fibers that form the stria of Gennari through layer IV of the human V1 is apparent at birth (Takashima et al., 1980), as are vertical connections between layers such as the apical dendrites of pyramidal cells drawn by Cajal (Figure 1Aii–iv). Horizontal connections emerge by layer, with long-range connections appearing in layers IV and V shortly before birth (Burkhalter et al., 1993). Those connections continue to mature postnatally, and the patchy arrangement of horizontal connections in superficial layers is found sometime after 4 months and becomes adult-like by 2 years (Burkhalter et al., 1993). Feedforward and feedback connections between V1 and V2 appear to mature sequentially, with feedforward developing by 4 months, but feedback connections continue to develop, similar to the maturation of horizontal connections in superficial layers (Burkhalter, 1993). Unfortunately, a limited number of anatomical studies with just a few cases have examined feedforward and feedback connections in human V1, so it is difficult to conclude how they develop.

### Postnatal development of human V1

Classic histological techniques have shown that anatomical structures of the human V1 are immature at birth. Still, some structures rapidly attain an adult-like appearance within the first few years of postnatal life (Conel, 1939, 1941, 1947, 1951, 1955, 1959, 1963, 1967; Huttenlocher et al., 1982; Burkhalter, 1993; Burkhalter et al., 1993). Cajal’s drawing of the infant V1 beautifully illustrates the cytoarchitecture of different laminae shortly after birth (Cajal, 1899). Although the infant (Figure 1Aii–iv) lacks the microanatomical complexity of the adult V1 (Figure 1Ai), it already has a dense network of neuronal processes that contribute to the appearance of the cortical layers. Nissl-stained sections through the occipital pole of the adult human cortex show the unique laminar appearance of V1, with the stria of Gennari in layer 4 distinguishing V1 from adjacent extra-striate areas (Figure 1B).

The adult-like appearance of layers in Nissl-stained sections from infants (Figure 1C) could be interpreted as evidence for early maturation of human V1. However, the primary function of Nissl-stained granular organelles in cells is the synthesis of proteins, which does not reflect all aspects of neuronal or glial maturity or plasticity. In contrast, molecular markers, especially for mechanisms that regulate experience-dependent plasticity, continue developing beyond the first few years of life.

Animal studies have shown that many neuronal and non-neuronal molecular mechanisms regulate experience-dependent plasticity in the visual system. It is beyond the scope of this mini-review to address all of those mechanisms, but there are excellent reviews that readers can use to gain a broader understanding (Huang et al., 1999; Berardi et al., 2003, 2004; Tropea et al., 2009; Takesian and Hensch, 2013; Pribiag and Stellwagen, 2014; Priebe and McGee, 2014; Sadahiro et al., 2016; Stephany et al., 2016; Hensch and Quinlan, 2018; Grieco et al., 2019; Benfey et al., 2022). Here, we focus on a set of glutamatergic and GABAergic mechanisms that are known to impact the role of excitatory and inhibitory neurotransmission in experience-dependent plasticity (Hensch, 2005; Hensch and Fagiolini, 2005; Maffei and Turrigiano, 2008; Smith et al., 2009; Tropea et al., 2009; Levelt and Hübener, 2012; Capogna et al., 2021) and have been well studied in human V1 across the lifespan (Murphy et al., 2005; Pinto et al., 2010, 2015; Williams et al., 2010; Siu et al., 2015, 2017; Siu and Murphy, 2018; Balsor et al., 2021).

### Animal studies−glutamatergic and GABAergic mechanisms regulating plasticity

This section reviews findings from animal studies to introduce the role of glutamatergic and GABAergic mechanisms in regulating experience-dependent plasticity in V1.

Most neurons and synapses in V1 are excitatory (glutamatergic), while only 20% of the neurons and an even smaller percentage of the synapses are inhibitory (GABAergic) (Beaulieu et al., 1992; Jones, 1993). The thalamocortical inputs to layer 4 are excitatory; however, many parvalbumin-positive (PV+) inhibitory interneurons are found in that layer (Balaram et al., 2014). Those PV+ neurons and processes modulate feedforward inputs to V1 (Pouille and Scanziani, 2001), thereby regulating experience-dependent plasticity (Sur et al., 2013; Capogna et al., 2021). This network of glutamatergic and GABAergic mechanisms contributes to shaping the maturation of V1 by guiding synapse development, transmitting visually-driven signals, and regulating experience-dependent plasticity to connect structure and function seamlessly.

Neurons expressing glutamatergic (NMDA and AMPA receptors) and GABAergic receptors are found in all layers of V1 (Albin et al., 1991; Hendry et al., 1994; Huntley et al., 1994; Trepel et al., 1998; Eickhoff et al., 2007, 2008), and developmental changes in those receptors contribute to experience-dependent plasticity. For example, NMDA receptors regulate how visual experience shapes the maturation of receptive field properties (e.g., ocular dominance, orientation tuning and direction selectivity) and the development of visual acuity (Kleinschmidt et al., 1987; Carmignoto and Vicini, 1992; Quinlan et al., 1999; Ramoa et al., 2001; Rivadulla et al., 2001; Fagiolini et al., 2003; Philpot et al., 2003, 2007; Smith et al., 2009). Also, GABAergic transmission and its receptors are essential for triggering the start and closure of the critical period and regulating the maturation of ocular dominance and orientation tuning in V1 (Tsumoto and Sato, 1985; Hensch et al., 1998; Huang et al., 1999; Fagiolini and Hensch, 2000; Morales et al., 2002; Fagiolini et al., 2004; Hensch, 2005). Together, glutamatergic and GABAergic mechanisms regulate the Excitatory-Inhibitory (E-I) balance. When GABAergic signaling is manipulated to shift that balance, it can either enhance or reduce visual plasticity depending on the age and in which GABAergic cells are engaged (Kirkwood and Bear, 1994; Iwai et al., 2003; Maffei et al., 2004; Hensch and Fagiolini, 2005). Importantly, even small changes in the E-I balance can dramatically alter plasticity in V1 (Fagiolini and Hensch, 2000).

NMDA receptors are key components regulating experience-dependent plasticity and are highly expressed prenatally in V1 when nascent synapses are functionally silent (Rumpel et al., 1998). NMDA receptors are tetrameric and contain two obligatory GluN1 subunits paired with two GluN2 (A-D) and/or GluN3 subunits (Monyer et al., 1994). The GluN2 subunits control the kinetics of the receptors. Nascent NMDA receptors have the slower GluN2B subunit, but visual experience drives the insertion of the faster GluN2A subunit into the receptor (Sheng et al., 1994). Furthermore, the GluN2A:GluN2B ratio controls the threshold for experience-dependent plasticity (Philpot et al., 2007; Yashiro and Philpot, 2008) and can be bidirectionally regulated by visual experience (Quinlan et al., 1999).

During the early stages of synapse development, few synapses express AMPA receptors, but visual-driven activity drives their insertion into nascent synapses, converting NMDAR-dominated silent synapses into active synapses (Rumpel et al., 1998). AMPA receptors are essential components of plasticity driven by long-term potentiation or homeostatic synaptic scaling (Gainey et al., 2009). Visual experience drives the accumulation of AMPA receptors at synapses to support visual responsiveness (Heynen et al., 2003; Lambo and Turrigiano, 2013), while deprivation leads to a loss of AMPA receptor expression in V1 (Williams et al., 2015).

Other glutamatergic mechanisms, such as the receptor scaffolding protein postsynaptic density 95 (PSD95), contribute to the experience-dependent refinement of V1. A mobile pool of PSD95 rapidly moves among active synapses, and that pool is dramatically reduced by sensory deprivation (Gray et al., 2006). Furthermore, a spike in PSD95 expression contributes to ending the critical period (Huang et al., 2015). Finally, PSD95, along with the scaffolding protein for GABA_A_ receptors, gephyrin, mediate the E-I balance (Prange et al., 2004; Lardi-Studler et al., 2007; Keith and El-Husseini, 2008) that is crucial for regulating experience-dependent plasticity in V1 (Hensch, 2004; Hensch and Fagiolini, 2005; Hensch and Quinlan, 2018).

The GABA_A_ receptor is the most abundant ionotropic GABA receptor in V1. More than 20 subunits combine to make a wide range of different GABA_A_ receptors (Sieghart, 1995; Cherubini and Conti, 2001) and three of those subunits, α1, α2 and α3, are developmentally regulated (Laurie et al., 1992; Hendrickson et al., 1994; Chen et al., 2001; Heinen et al., 2004). Nascent GABA_A_ receptors have more α2 and α3 subunits, but during development, there is a shift to more α1 and a faster inhibitory postsynaptic potential (IPSP) (Gingrich et al., 1995). The α1 subunit is the high-affinity interface for binding the neurotransmitter GABA and benzodiazepines, so its development has a crucial role in GABAergic neurotransmission (Pritchett et al., 1989; Smith and Olsen, 1995; Sigel, 2002). Abnormal visual experience during the critical period accelerates the developmental shift to more GABA_A_α1 (Beston et al., 2010; Balsor et al., 2019) while GABAergic synapses containing α2 receptors regulate neuronal firing (Fagiolini et al., 2004). However, only α1-containing receptors drive experience-dependent critical period plasticity (Fagiolini et al., 2004).

In the next section, we review the pattern of developmental changes in these glutamatergic and GABAergic mechanisms in human V1 and highlight insights from those developmental trajectories that may influence the potential for experience-dependent plasticity.

### Human studies−development of glutamatergic and GABAergic mechanisms

Animal studies have shown that the development of glutamatergic and GABAergic mechanisms regulates many aspects of experience-dependent plasticity in V1. Moreover, in human V1, magnetic resonance spectroscopy has been used to observe changes in glutamate and GABA linked to visual stimulation, age and plasticity (Lin et al., 2012; Lunghi et al., 2015; Pitchaimuthu et al., 2017; Wijtenburg et al., 2017; Kurcyus et al., 2018; Frank et al., 2022). Developmental transcriptome databases like the BrainSpan (Miller et al., 2014) contain information about those mechanisms, but only a few studies provide analyses of postnatal changes in human V1 (Zhu et al., 2018; Balsor et al., 2021; Natu et al., 2021). Our laboratory has a library of postmortem tissue samples from human V1 that we have used to explore the postnatal development of many glutamatergic, GABAergic proteins and a select few other plasticity-related proteins. Those studies are included in the review below.

**FIGURE 2:**
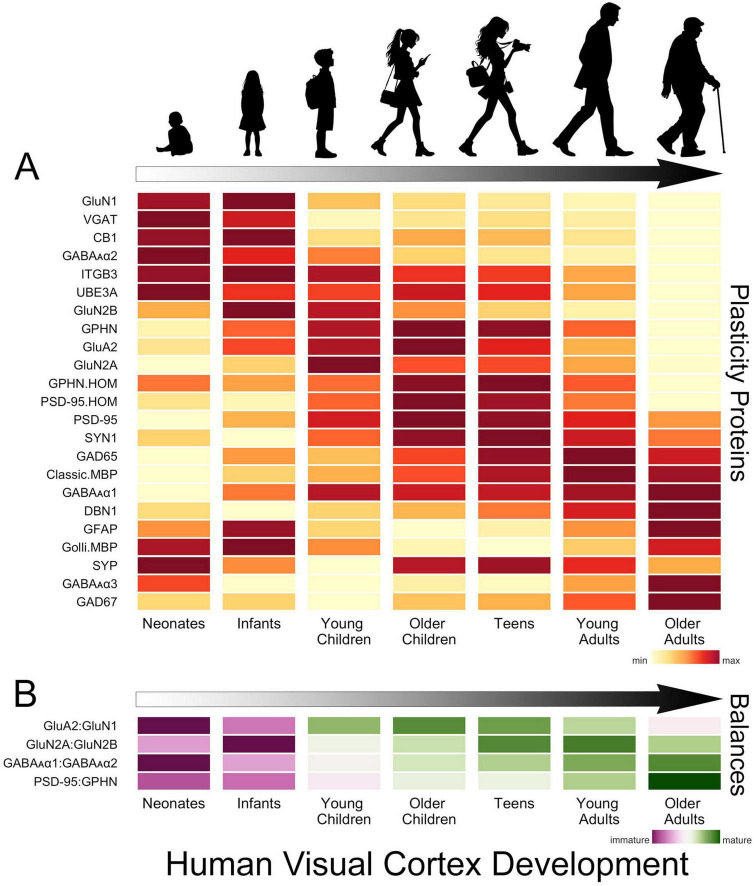
Development of plasticity proteins and balances in human V1. **(A)** The heatmap shows the development of a set of glutamatergic, GABAergic and a select few other plasticity-related proteins in human V1. The colors indicate the development of each protein across the lifespan, where yellow is the lowest and dark red is the highest expression level. There are waves of high expression (dark red) in each stage, highlighting multiple timescales for the development of these plasticity proteins. **(B)** The heatmap shows the development of a set of balances known to regulate experience-dependent plasticity in V1. The colors reflect maturation, with the purplish colors indicating that the earlier developing protein is dominant and greenish that the later developing protein dominates the balance. The proteins that develop earlier (GluN1, GluN2B, GABA_A_α2) dominate the balances in neonates and infants, but all of the balances shift toward the later developing proteins (GluA2, GluN2A, GABA_A_α1, PSD95) in childhood. The GluA2:GluN1 balance matures rapidly in childhood, but the other balances continue to mature slowly into teens, young adults and older adults. The time course of changes in these balances is significant as they indicate lifelong changes in the mechanisms regulating plasticity in human V1. The data used to create these figures are from the following papers (Murphy et al., 2005; Pinto et al., 2010, 2015; Williams et al., 2010; Siu et al., 2015, 2017).

In human V1, the development of glutamatergic and GABAergic molecular mechanisms proceeds in waves across multiple timescales that cover the lifespan (Murphy et al., 2005; Pinto et al., 2010, 2015; Williams et al., 2010; Siu et al., 2017; Siu and Murphy, 2018; Figure 2A). The obligatory subunit of NMDA receptors, GluN1, is highly expressed at birth in human V1, then quickly declines over the first 2 years of life (Murphy et al., 2005; Siu et al., 2017; Siu and Murphy, 2018). That loss is balanced by an increase in AMPA receptors (e.g., GluA2) and the maturational shift from more NMDA (GluN1) in infancy to more AMPA receptors in older children (Murphy et al., 2005; Siu et al., 2017; Siu and Murphy, 2018). The rapid response properties of AMPA receptors imbue synapses with strong feedforward responses to visually driven activity, while the slower NMDA receptors contribute to feedback modulation of visual processing in adult V1 (Self et al., 2012). So the early shift in the AMPA:NMDA (GluA2:GluN1) balance suggests the rapid establishment of feedforward connections to human V1, followed by an extended period when feedback and other connections are established (Figure 2B).

Through infancy and young childhood, the GluN2B subunit dominates the composition of NMDA receptors in V1, and there is little expression of GluN2A or the receptor anchoring protein PSD95 (Murphy et al., 2005; Pinto et al., 2015; Siu et al., 2017; Siu and Murphy, 2018; Figure 2A). GluN2B declines through childhood to stable levels in adolescence while expression of PSD95 peaks in older childhood when the period for susceptibility to amblyopia ends. However, GluN2A does not dominate the GluN2A:GluN2B balance until teens to young adults (Siu et al., 2017; Siu and Murphy, 2018; Figure 2B). The gradual decline of GluN2B and subsequent increase of GluN2A suggests a prolonged period of NMDA-dependent plasticity in human V1 that may impact the threshold for synaptic modification (Philpot et al., 2007; Smith et al., 2009) and ocular dominance plasticity (Cho et al., 2009). Furthermore, in older adults, the loss of GluN2A triggers a shift in the GluN2A:GluN2B balance back to the pattern observed in childhood (Siu et al., 2017), but age-related changes in other mechanisms suggest this is not a return to child-like plasticity.

Developmental changes in GABAergic mechanisms over the first few years include loss of the presynaptic proteins VGAT and cannabinoid receptor CB1 and the postsynaptic receptor subunits GABA_A_α2 and GABA_A_α3. Over those same ages there is a gradual increase in expression of the postsynaptic receptor subunit GABA_A_α1 (Murphy et al., 2005; Pinto et al., 2010; Siu and Murphy, 2018; Figure 2A). The early loss of GABA_A_α2 causes a shift in favour of the α1 subunit (Figure 2B), which is important because the α1 subunit is necessary to engage experience-dependent plasticity in V1 (Fagiolini et al., 2004). In monkey V1, the shift from more α2 to more α1 is completed during early development (Hendrickson et al., 1994). However, in humans, the expression of α2 continues to decline across the lifespan, and the shift to α1 is more gradual (Pinto et al., 2010). Other GABAergic mechanisms also have prolonged development into older childhood, adolescence and adulthood. Gephyrin (GPHN) anchors GABA_A_ receptors at the synapse, regulates the number of excitatory and inhibitory synapses, GABAergic synapse plasticity and the physiological E-I balance (Prange et al., 2004; Lardi-Studler et al., 2007; Keith and El-Husseini, 2008; Tyagarajan and Fritschy, 2014). Gephyrin peaks from adolescence to adulthood before declining into aging (Pinto et al., 2010). The enzyme that makes the basal pool of GABA, GAD67, is relatively constant, but the enzyme that makes the on-demand pool of GABA, GAD65, increases gradually until young adulthood before declining during aging of human V1 (Pinto et al., 2010). Together, these identify both pre- and post-synaptic losses of GABAergic signaling in aging human V1. These losses may contribute to changes in visual perception (Owsley, 2011) since the age-related loss of GABA in monkey V1 underlies poor orientation tuning (Leventhal et al., 2003).

There are lifelong changes in the expression of PSD95 and gephyrin (GPHN), two of the mechanisms regulating the E-I balance (Keith and El-Husseini, 2008). In infancy, gephyrin dominates the E-I balance (PSD95:GPHN), followed by a gradual shift to roughly equal expression of PSD95 and gephyrin in adults, then in aging, a shift back to more gephyrin (Figure 2B). The changes in the ratio of PSD95:gephyrin suggest that the E-I balance is continuously shifting and changing its contribution to plasticity in human V1 across the lifespan (Figure 2B).

Developmental changes in the presynaptic proteins synapsin (SYN1) and synaptophysin (SYP) and the dendritic spine marker drebrin A (DBN1) provide additional insights into synapse development in human V1. Both synapsin and synaptophysin change through infancy and childhood but in opposite ways; synapsin increases while synaptophysin decreases through infancy before rebounding in older childhood. However, neither of these synapse markers follows the trajectory described by electron microscopy studies that counted synapses (Huttenlocher et al., 1982). Furthermore, drebrin (DBN1), which is found in dendritic spines at excitatory synapses and supports homeostatic plasticity (Aoki et al., 2009; Takahashi and Naito, 2017), gradually increases across the lifespan, pointing to lifelong synaptic changes i human V1.

The development of a few other plasticity-related proteins has been studied in human V1. One of these mechanisms is the E3 ubiquitin ligase UBE3A, which controls ARC’s degradation and regulates AMPA receptor internalization (Greer et al., 2010). Knocking out UBE3A in mouse V1 leads to a loss of experience-dependent plasticity, leaving connections rigid and unable to be fine-tuned by visual experience (Yashiro et al., 2009). In human V1, the expression of UBE3A is highest in infancy, with a smaller peak in older childhood and adolescence, followed by a steady decline into aging (Williams et al., 2010). Another mechanism that regulates the expression of AMPA receptors, beta3 integrins (ITGB3) (Cingolani et al., 2008; McGeachie et al., 2011), has a pattern of expression similar to UBE3A, suggesting that age-related declines in these mechanisms may lead to endocytosis of AMPA receptors that reduce synaptic scaling plasticity. In addition, a glial mechanism known to reduce experience-dependent plasticity, cortical myelin (classic myelin basic protein MBP) (McGee et al., 2005), gradually increases in human V1 into the fourth decade of life (Siu et al., 2015). These 3 proteins contribute to the brakes on plasticity, and their prolonged changes suggest a gradual increase in the brakes and loss of the potential for experience-dependent plasticity in human V1.

Figure 2 summarizes the changes in the proteins and balances across the lifespan. It is clear that this collection of plasticity-related proteins has peaks at different ages across the lifespan (Figure 2A). The balances also show this pattern of both early (GluA2:GluN1) and prolonged changes [E-I (PSD95:GPHN)]. These findings underscore that multiple timescales and gradual shifts in plasticity mechanisms across the lifespan characterize human V1 development.

## Discussion

This review covers prenatal and postnatal development of anatomical and molecular features in human V1. Studies using various neurobiological techniques show that human V1 develops and changes over multiple timescales from the first trimester to aging, so whether human V1 is described as developing early or later depends on what is studied. Importantly, prolonged changes are found for many of the proteins that regulate plasticity, suggesting that some forms of experience-dependent plasticity in human V1 extend past childhood. Only a handful of plasticity mechanisms have been studied, so much is still unknown about the neural mechanisms that regulate plasticity in human V1.

One aspect of human V1 development that has not been explored is the impact of host factors, such as sex, genetic ancestry, and socioeconomic status. However, brain banks have small numbers of cases with limited diversity, making it challenging to do those studies. Still, it is crucial to understand the impact of host factor diversity on human V1 since those factors may account for developmental variability and the timing of plasticity.

The methods used to study the anatomy and molecular environment of human V1 continue to identify new features of its development. Molecular tools have revealed distinct aspects of human V1 (Kang et al., 2011; Carlyle et al., 2017; Jorstad et al., 2023; Wang et al., 2024) and its relationship to neurodevelopmental disorders (Gandal et al., 2022). The next wave of single-cell and spatial transcriptomic tools is poised to uncover new details about how the human cortex develops (Velmeshev et al., 2023; Qian et al., 2024). We end with a reminder from Cajal’s words about the importance of the methods for studying human V1:

*The minute anatomy of the visual cortex (region of the calcarine fissure, sulcus lobulus lingualis) has already been explored by several investigators, among whom we may make particular mention of Meynert et al. But their very incomplete researches have been performed by such insufficient methods as staining with carmine, the Weigert-Pall method, or that of Nissl with basic anilines – methods which, as is well known, do not suffice at all to demonstrate the total morphology of the elements and the organization of the most delicate nerve plexuses (Cajal, 1899)*.
